# Renal hemodynamics, function, and oxygenation during cardiac surgery performed on cardiopulmonary bypass: a modeling study

**DOI:** 10.14814/phy2.12260

**Published:** 2015-01-19

**Authors:** Ioannis Sgouralis, Roger G. Evans, Bruce S. Gardiner, Julian A. Smith, Brendan C. Fry, Anita T. Layton

**Affiliations:** Department of Mathematics, Duke University, Durham, North Carolina; Department of Physiology, Monash University, Clayton, Victoria, Australia; Department of Computer Science and Software Engineering, University of Western Australia, Crawley, Western Australia, Australia; Department of Surgery, Monash University, Clayton, Victoria, Australia

**Keywords:** Hypoxia, kidney, medulla, metabolism

## Abstract

Acute kidney injury, a prevalent complication of cardiac surgery performed on cardiopulmonary bypass (CPB), is thought to be driven partly by hypoxic damage in the renal medulla. To determine the causes of medullary hypoxia during CPB, we modeled its impact on renal hemodynamics and function, and thus oxygen delivery and consumption in the renal medulla. The model incorporates autoregulation of renal blood flow and glomerular filtration rate and the utilization of oxygen for tubular transport. The model predicts that renal medullary oxygen delivery and consumption are reduced by a similar magnitude during the hypothermic (down to 28°C) phase of CPB. Thus, the fractional extraction of oxygen in the medulla, an index of hypoxia, is increased only by 58% from baseline. However, during the rewarming phase (up to 37°C), oxygen consumption by the medullary thick ascending limb increases 2.3‐fold but medullary oxygen delivery increases only by 33%. Consequently, the fractional extraction of oxygen in the medulla is increased 2.7‐fold from baseline. Thus, the renal medulla is particularly susceptible to hypoxia during the rewarming phase of CPB. Furthermore, autoregulation of both renal blood flow and glomerular filtration rate is blunted during CPB by the combined effects of hemodilution and nonpulsatile blood flow. Thus, renal hypoxia can be markedly exacerbated if arterial pressure falls below its target level of 50 mmHg. Our findings suggest that tight control of arterial pressure, and thus renal oxygen delivery, may be critical in the prevention of acute kidney injury associated with cardiac surgery performed on CPB.

## Abstract (precis)

Open heart surgery saves lives, but unfortunately, up to 30% of patients are left with injured kidneys, with the worst (~1%) cases, patients requiring lifelong dialysis therapy or a kidney transplant. Hypoxia in the inner part of the kidney (the medulla) is thought to occur during cardiopulmonary bypass and so drive the development of kidney injury. Using a computational model, we conducted simulations that suggest that the medulla is particularly susceptible to hypoxia during the period patients are rewarmed at the end of the operation, before they are weaned from cardiopulmonary bypass.

## Introduction

Acute kidney injury (AKI) is a prevalent complication of cardiac surgical procedures that require cardiopulmonary bypass (CPB) (Karkouti et al. 2009). Even mild AKI following CPB cardiac surgery is prognostically important, being associated with a more than fourfold increase in the risk of in‐hospital death (Karkouti et al. 2009) as well as extended hospital stays through additional complications (Karkouti et al. 2009). When AKI is severe enough to require renal replacement therapy (in 1–2% of patients who have undergone cardiac surgery under CPB), mortality rate exceeds 60% (Mangano et al. 1998).

Renal hypoxia might be an important pathway in the development of AKI during CPB cardiac surgery, particularly if there is a mismatch between changes in renal oxygen delivery and oxygen consumption (Evans et al. 2013). Renal oxygen delivery is mainly determined by renal blood flow (Evans et al. 2013). Renal oxygen consumption is mainly driven by the metabolic work of tubular sodium reabsorption, which in turn is largely driven by the filtered load of sodium, and thus glomerular filtration rate (GFR) (Evans et al. 2014). Mean arterial pressure is often set to a low level during CPB (50–70 mmHg), and further, autoregulation of renal blood flow and GFR is compromised (Andersson et al. 1994). Consequently, changes in renal blood flow and GFR during CPB will likely have a major impact on the risk of AKI.

Multiple factors compromise renal autoregulation during CPB. Firstly, the CPB circuit must be primed with a cell‐free solution, resulting in hemodilution (Rosner et al. 2008). The potential importance of hemodilution is evidenced by the observation that a hematocrit of less than 21% is an independent risk factor for AKI after cardiac surgery (Rosner et al. 2008). Secondly, tissue perfusion during CPB is nonpulsatile. The pulsatility of renal arterial pressure is a critical determinant of the myogenic component of the autoregulatory response, as evidenced by experimental findings and simulations from mathematical models (Loutzenhiser et al. 2002; Chen et al. 2009; Sgouralis and Layton 2012). Collectively, these data indicate that oscillations in renal arterial pressure induced by the beating heart lead to sustained vasoconstriction of the afferent arteriole.

Thus, it seems reasonable to hypothesize that renal ischemia could arise during CPB as a result of complex interactions between the effects of altered blood viscosity due to hemodilution and hypothermia, the absence of pulsatility of blood flow, and hypotension. Clearly, our ability to study these phenomena in the clinical situation is limited. Our ability to study them in intact animals is also limited, because of the number of experimental conditions that would need to be met in order to allow such interactions to be fully interrogated. Therefore, in the current study we have utilized a computational model to examine how the renal hemodynamic changes during CPB might lead to renal circulatory dysfunction and hypoxia, and determine the phase during CPB in which the kidney is most vulnerable to hypoxic injury. Because anatomic data on the human kidney are limited, baseline model parameters are based on the rat, whose kidney is the most well studied, whereas clinical data from human patients are used to model the effects of CPB.

## Mathematical Model

**Figure 1.**
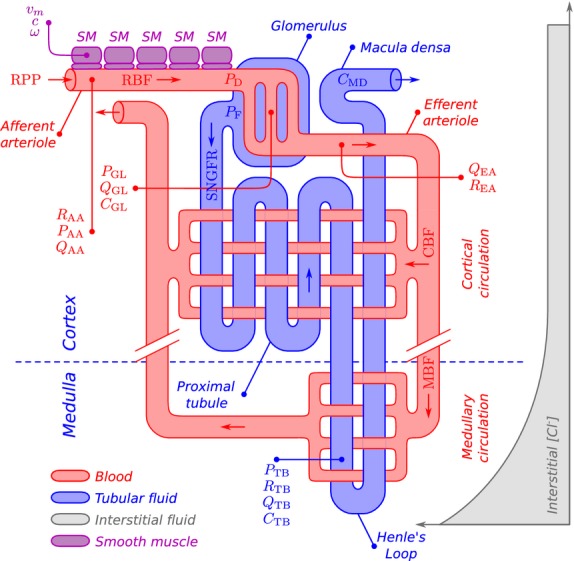
Schematic representation of the renal model. Blood is supplied through the afferent arteriole. In the glomerulus, a portion of the delivered plasma is directed to the proximal tubule, and the rest to the efferent arteriole. Cortical and medullary capillary beds are supplied by the efferent arteriole outflow. The afferent arteriole is shown with five smooth muscles (SM), whereas in the model 101 SM are represented.

To model hemodynamic control and oxygenation in the kidney, we have extended a mathematical model, previously developed by us (Sgouralis and Layton 2014), which represents the functional unit of the kidney: a nephron with the supplying vessel. Specifically, the model consists of (1) an afferent arteriole; (2) a glomerulus; (3) a nephron. A schematic diagram of the model is shown on Figure 1. Briefly, the afferent arteriole delivers blood to the glomerulus. A portion of the plasma is filtered into the nephron, where fluid and sodium reabsorption takes place. The remaining blood is drained to the efferent arteriole and then distributed to either the cortex or medulla.

### Autoregulatory mechanisms

Renal autoregulation is mainly mediated by the myogenic response and tubuloglomerular feedback, both of which regulate single nephron glomerular filtration rate (SNGFR) by inducing constriction or dilation of the afferent arteriole (Holstein‐Rathlou and Marsh 1994; Cupples and Braam 2007; Just 2007). With the myogenic response, a rise in intravascular pressure elicits a reflex constriction that generates a compensatory increase in vascular resistance. Tubuloglomerular feedback is a negative feedback response that balances glomerular filtration with tubular reabsorptive capacity.

### Renal blood flow

The afferent arteriole is the effector of the autoregulatory mechanisms. To properly simulate those mechanisms, the model afferent arteriole represents detailed ionic transport and muscle mechanics of a series of arteriolar smooth muscle cells, coupled electrically. Each arteriolar smooth muscle cell model incorporates cell membrane potential, transmembrane ionic transport, cytosolic Ca^2+^ regulation, and muscle contraction. Key model equations can be found in the Appendix 1. Vascular flow is described as quasi‐steady Poiseuille flow. Thus, given renal perfusion pressure (RPP), the arteriolar model predicts blood flow Q_AA_.

### Glomerular filtration

The model glomerulus is represented as a single capillary, connected between the afferent and efferent arterioles. Model equations are based on conservation of plasma and protein. Plasma filtration is characterized by the ultrafiltration coefficient *K*_f_ (see Appendix 1). Given arteriolar outflow *Q*_AA_, the glomerulus model predicts SNGFR. We assume that 90% of the efferent arteriolar flow is directed to the cortex, and the remaining 10%, denoted *f*^med^, to the medulla. Thus, the flow of red blood cells to the medulla is given by 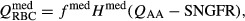
where *H*^med^ denotes the medullary hematocrit, which is 20% lower than systemic hematocrit (Zimmerhackl et al. 1985). Plasma flow is given by 
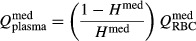
Oxygen delivery to the medulla (denoted 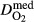) is given by the total amount of free and hemoglobin‐bound oxygen (Popel 1989) 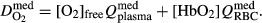


### Tubular transport

The model nephron comprises a proximal tubule and a short loop of Henle. The model predicts intratubular pressure, water flow, and tubular fluid [Cl^−^], which is the key signal to tubuloglomerular feedback (see below). Tubular fluid [Cl^−^] is given by conservation of mass 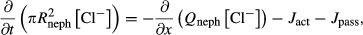
where *R*_neph_ is tubular luminal radius, which is assumed to vary passively with luminal fluid pressure, and *Q*_neph_ is the tubular fluid flow. *J*_act_ and *J*_pass_ denote the active and passive transport of Cl^−^, respectively, assumed positive into the tubule 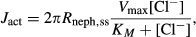


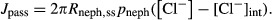


*R*_neph,ss_ denotes the steady‐state radius. Active transport is assumed to be characterized by Michaelis–Menten kinetics, which is an approximation for the membrane transport processes that include the cotransport of Na^+^, K^+^, and (two) Cl^−^ ions by the apical NKCC2 transporter, the active transport by Na^+^ ion via the basolateral Na^+^/K^+^‐ATP pump, and the passive diffusion of the Cl^−^ ion through the basolateral membrane. Passive transepithelial diffusion is characterized by permeability *p*_neph_. Interstitial [Cl^−^], denoted [Cl^−^]_int_, is assumed known a priori.

Based on Na^+^/K^+^‐ATPase stoichiometry, which suggests that at maximum efficiency, 18 moles of Na^+^ are transported per mole of O_2_ consumed, we compute the rate of O_2_ consumption due to active transport by *J*_act_/18. Thus, total O_2_ consumption by the medullary structures, given per nephron, is determined by summing the total O_2_ consumption rate by the medullary segments (i.e., proximal straight tubule and, most importantly, the medullary thick ascending limb) and the basal consumption, that is, 
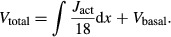


The integral is computed over the medullary segments only. *V*_basal_ denotes basal consumption per nephron, that is, total basal O_2_ consumption in the medulla, divided by the number of nephrons. Medullary basal O_2_ consumption is set to 0.70 pmol/min/nephron, based on (Fry et al. 2014).

## Results

### Impact of hypothermia

We first studied the effects of temperature on renal function by simulating two non‐CPB scenarios, normothermia (base case), and hypothermia, in which body temperature is assumed to be 37°C and 28°C, respectively. Key model parameters for these cases are summarized in Table 1. A comparison between renal function predicted in the normothermic and hypothermic cases indicate the substantial effect of hypothermia on blood flow and oxygenation (Table 2). A comparison between model predictions and experimental findings reported in (Broman and Källskog 1995) validates the model.

**Table 1.** Parameter values for the simulated cases. The normothermic and hypothermic cases are motivated by (Broman and Källskog 1995), and the CPB phases are motivated by (Andersson et al. 1994).

	Normothermia	Hypothermia	Pre‐CPB	CPB‐hypothermia	CPB‐rewarming	Post‐CPB	Units
Renal perfusion pressure	120	120	75	50	50	75	mmHg
Pulse amplitude	20	20	20	0	0	20	mmHg
Systemic hematocrit	45	45	45	25	25	25	%
Body temperature	37	28	37	28	37	37	°C
Arterial PO_2_	100	100	100	400	400	100	mmHg

**Table 2.** Comparison of simulated normo‐ and hypothermia. Renal blood flow, Henle's loop flow, nL/min/nephron; SNGFR, nL/min; glomerular blood pressure (BP), Bowman space pressure, mmHg; net NaCl reabsorption, pmol/min/nephron.

			Change (%)	
	Normothermia	Hypothermia	Model	Ref. (Broman and Källskog 1995)
Renal blood flow	290.3	156.6	−46.1	−44.5
SNGFR	30.0	15.8	−47.4	−49.1
Glomerular BP	50.7	45.0	−11.3	−18.2
Bowman pressure	14.0	13.9	−0.5	−2.5
Henle's loop flow	8.8	4.6	−48.4	−49.3
Net reabs.	3048	1577	−48.3	−49.3

The model represents the following temperature‐dependent phenomena. For simplicity, model parameters are fitted as linear functions to the available data at 37°C and 28°C.

1. We assume that *afferent arteriole myocyte cytosolic* [Ca^2+^] increases at lower temperature. This is achieved by decreasing afferent arteriole smooth muscle cytosolic Ca^2+^ extrusion rate with temperature (Broman and Källskog 1995).
2. We assume that *efferent arteriole muscle activation* increases with temperature (Broman and Källskog 1995).
3. *Plasma and tubular fluid viscosities* increase with decreasing temperature (Broman and Källskog 1995; Lim et al. 2010).
4. The *ultrafiltration coefficient* K_f_ decreases with decreasing temperature (Broman and Källskog 1995).
5. Broman and Källskog (Broman and Källskog 1995) reported GFR, urine flow and composition produced by groups of rats with body temperate kept at 37°C and 28°C, respectively, and whose kidneys were moderately concentrating. Based on those data, we assume that *thick ascending limb maximum transport rate V*_max_ decreases with decreasing temperature. Model formulation (Appendix 1 Eq. 44) assumes zero time lag between the increase in temperature and the adjustment of active tubular transport.

**Figure 2.**
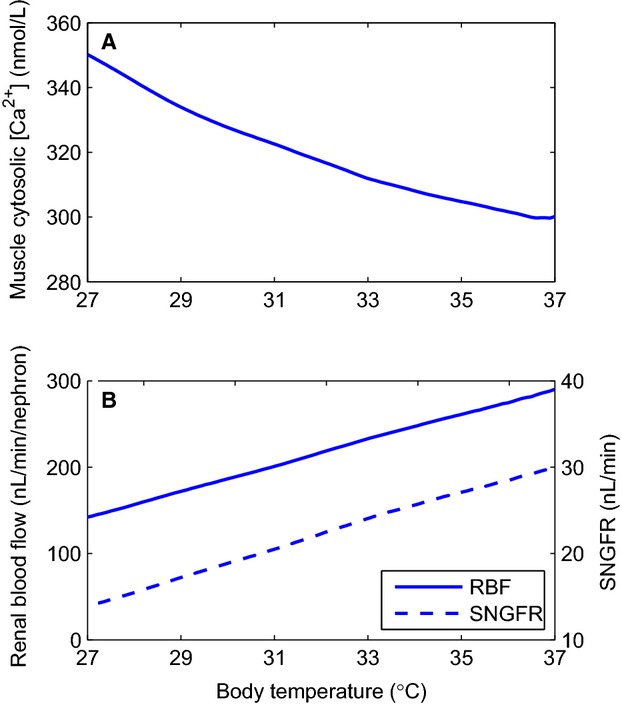
Afferent arteriole response to temperature changes. Panel A, average myocyte cytosolic [Ca^2+^]; panel B, renal blood flow, and SNGFR. Temperature decline increases cytosolic [Ca^2+^], which leads to vasoconstriction and reduction in blood flow and SNGFR.

In large part owing to the slower Ca^2+^ extrusion rate at low temperatures, smooth muscle cytosolic [Ca^2+^] increases with decreasing temperature; see Figure 2A. The elevated cytosolic [Ca^2+^] leads to vasoconstriction and reduction in blood flow and SNGFR; Figure 2B. The observed temperature mediated reduction in blood flow and SNGFR is further amplified by the rise in blood viscosity and decrease in glomerular filtration coefficient.

At 28°C, the model predicts a marked 46.1% decrease in arteriolar flow, which results in a 47.4% reduction in SNGFR. In addition to its effect on blood flow, lower temperature also reduces thick ascending limb active transport. As a result, the model predicts a 48.3% lower NaCl reabsorption rate in the hypothermic case. This yields an elevated luminal [Cl^−^] at the macula densa (53.0 vs. 46.1 mmol/L), which activates tubuloglomerular feedback and further reduces SNGFR. The relative changes reported above are in good agreement with the measurements of Broman and Källskog (Broman and Källskog 1995) (Table 2).

### Renal function and hemodynamics during CPB

In the next set of simulations, we aimed to identify the phase in CPB when the kidney is most vulnerable to hypoxic injury. Four CPB phases were considered: (1) pre‐CPB, defined as the period following induction of anesthesia and prior to initiation of CPB; (2) hypothermic CPB, the hypothermic period of the surgery; (3) CPB rewarming, the normothermic period of the surgery; and (4) post‐CPB, the post‐surgery period in which the effects of hemodilution and anesthesia persist. Model parameters for these cases are given in Table 1.

Besides body temperature (lower in hypothermic CPB), hemodynamics also differ substantially among the four CPB phases.

1. Notably, due to the effects of anesthesia, RPP is lower than baseline in all four phases, but particularly so during the hypothermic CPB and CPB rewarming phases.
2. During the pre‐ and post‐CPB phases, blood circulation is driven by the heart. The resulting pulsatile flow is modeled by
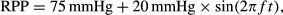
where the frequency *f* is set to 1 Hz and t is given in seconds. The CPB pump used during surgery does not generate pulsatile flow. Thus, RPP is assumed constant at 50 mmHg for the hypothermic CPB and CPB rewarming phases.
3. Except for the pre‐CPB phase, systemic hematocrit is substantially lower than normal. A lower hematocrit results in a lower effective blood viscosity (Pries et al. 1992; Pries and Secomb 2003), which the model accounts for by incorporating the empirical hematocrit–viscosity relation obtained by Pries et al. in (Pries et al. 1994) (equation (9) therein).
4. The impact of hemodilution on oxygen delivery is partially compensated by the ventilation of the patient with almost 100% oxygen during the hypothermic CPB and CPB rewarming phases.

**Figure 3.**
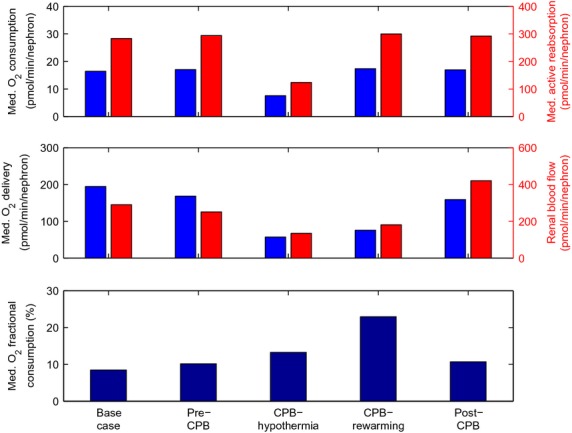
Medullary function and oxygenation during CPB. Model predicts reduced medullary oxygenation during the hypothermic and rewarming phases of CPB. The reduction is significantly pronounced during rewarming.

**Figure 4.**
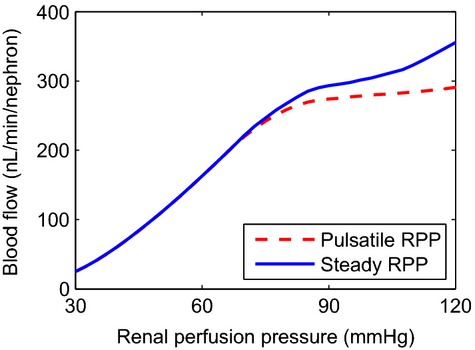
Time‐averaged blood flow for pulsatile and steady RPP. SNGFR exhibit similar trends. Autoregulation is blunted when flow is nonpulsatile. Nonetheless, in the range of arterial pressure values relevant to CPB, both cases produce similar blood flow.

Key renal function and hemodynamic predictions are summarized in Table 3 and Figure 3. The pre‐CPB phase differs from baseline only in a lower RPP (75 vs. 100 mmHg). The lower RPP triggers a myogenic response that induces vasodilation which stabilizes renal blood flow and SNGFR. The effectiveness of the model myogenic response can be seen in Figure 4, which shows predicted time‐averaged blood flow for a range of mean arterial pressures, obtained for pulsatile and steady RPP. The model predicts efficient autoregulation between 80 and 115 mmHg when RPP is pulsatile, which is somewhat blunted when RPP is nonpulsatile. Given a RPP of 75 mmHg during the pre‐CPB phase, the model predicts 13.4% and 6% reductions in blood flow and SNGFR.

**Table 3.** Summary of renal function during CPB. Renal blood flow, nL/min/nephron; SNGFR, nL/min; medullary active NaCl reabsorption, O_2_ delivery, O_2_ consumption, pmol/min/nephron.

	Base case	Pre‐CPB	CPB‐hypothermia	CPB‐rewarming	Post‐CPB
Renal blood flow	290.3	251.2	134.2	180.9	420.3
SNGFR	30.0	28.2	11.6	17.2	30.9
Filtration %	19.0	21.1	11.5	12.7	10.1
Active reabs.	283.1	294.4	123.7	299.7	292.3
O_2_ delivery	194.5	168.2	57.1	75.7	158.8
O_2_ consumption	16.4	17.1	7.6	17.4	16.9
Consumption %	8.4	10.1	13.3	22.9	10.7

Hemodilution begins during the hypothermic phase of CPB. Taken in isolation, the lower blood viscosity at a lower hematocrit would increase blood flow. However, RPP is further reduced to 50 mmHg, which, as seen in Figure 4, falls below the range of RPP where autoregulation can sufficiently compensate for the pressure variations and stabilize SNGFR. Furthermore, temperature is decreased to 28°C. As shown in the preceding set of simulations, hypothermia markedly decreases blood flow and SNGFR. Together, these factors result in a 53.8% reduction in arteriolar blood flow, and a 61.3% reduction in SNGFR. The drastically lowered medullary blood flow (MBF) and hemodilution decrease medullary oxygen delivery, which is only partially compensated for by the fourfold increase in blood PO_2_. Consequently, medullary oxygen delivery is only 29.4% that of the base case, its minimum level of all the phases of CPB.

With the transition to the rewarming phase of CPB, body temperature is restored to 37°C, resulting in substantially greater renal blood flow and SNGFR compared to the hypothermic phase, although those flows remain substantially lower than in the base case. (Body temperature likely increases gradually from 28°C to 37°C; however, here we consider only the final body temperature of 37°C.) A particularly notable effect of a higher body temperature is the increase in thick ascending limb active transport. Consequently, medullary O_2_ consumption is 129% greater than in the hypothermic phase of CPB. Thus, even though medullary oxygen delivery is greater in the rewarming phase of CPB than the hypothermic phase, the rewarming phase is associated with a greater medullary fractional O_2_ extraction (22.9%, compared to 13.3% in the hypothermic phase of CPB and 8.4% in the base case).

In the post‐CPB phase, RPP, and blood PO_2_ are restored to the pre‐CPB levels, but the effects of hemodilution persist. The model predicts that, owing to the lower hematocrit and blood viscosity, renal blood flow is elevated, but the other renal functions are not significantly different from the base case. Notably, despite a low hematocrit and baseline (not increased) blood PO_2_, medullary O_2_ supply is only slightly lower, and medullary O_2_ consumption is only slightly higher, than the base case. The predicted PO_2_ at different CPB phases exhibit trends that are consistent with a pilot study in pigs (Stafford‐Smith and Grocott 2005).

### Impact of variations in surgical conditions

**Figure 5.**
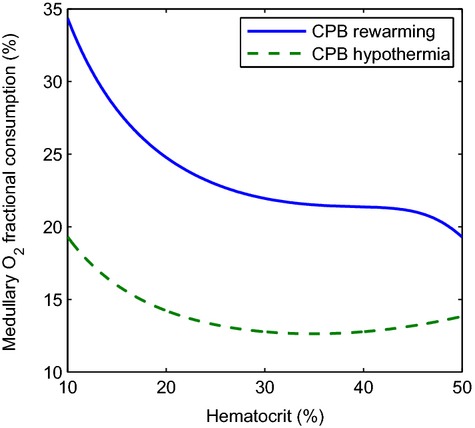
Response of renal oxygenation to hematocrit changes during the CPB hypothermic and rewarming phases. A sharp decline in hematocrit leads to significant increase in medullary O_2_ fractional consumption. The effect is significantly more prominent during rewarming.

During each phase of the CPB surgery, hematocrit likely fluctuates substantially. To determine the effects on renal oxygenation, we simulated the CPB hypothermic and rewarming phases for a range of hematocrit values, and computed the resulting O_2_ fractional extraction. Simulation results, shown in Figure 5, indicate that large declines in hematocrit yield substantial increases of O_2_ consumption, with the effect being significantly more prominent during the rewarming phase.

The interstitial [NaCl] axial gradient along the outer medulla is generated primarily by the metabolically driven Na^+^ reabsorption from the thick ascending limb. At low temperatures, Na^+^ reabsorption is slowed, which may lower the interstitial [NaCl]. To assess the model's response to changes in interstitial [NaCl], we simulated the CPB hypothermic and rewarming phases with interstitial [Cl^−^] reduced by 50%. That is, interstitial [Cl^−^] increases from 115 mmol/L at the corticomedullary boundary to 195 mmol/L (compared to 275 mmol/L in the base case) at the outer–inner medullary boundary. Key predictions, shown on Table 4, suggest that model results are mostly insensitive to changes in interstitial [NaCl], owing, in large part, to the small permeability of the thick ascending limbs (1.5 × 10^−5^ cm/sec).

**Table 4.** Renal oxygenation response to interstitial [Cl^−^] gradient drops during the CPB hypothermic and rewarming phases. Interstitial [Cl^−^], mmol/L; renal blood flow, nL/min/nephron; medullary active NaCl reabsorption, pmol/min/nephron.

	CPB‐hypothermia		CPB‐rewarming	
Max. [Cl^−^]_int_	275	195	275	195
Renal blood flow	134.2	134.2	180.9	180.9
Active reabs.	123.7	114.1	299.7	275.9
Consumption %	13.3	12.3	22.9	21.2

Having identified rewarming as the CPB phase with the largest fractional medullary O_2_ consumption, and thus the highest degree of vulnerability to renal hypoxic injury, we consider the effects on renal function of varying RPP, hematocrit, and the degree of MBF autoregulation during rewarming. In volume‐expanded rats, MBF has been observed to be autoregulated to a lesser extent than renal blood flow (Fenoy and Roman 1991; Mattson et al. 1993). To incorporate that finding, we modify Equation 1 as follows 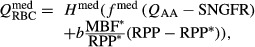
where *b* controls the degree of MBF autoregulation and the asterisks denote reference values. In the base case, *b* is set to 0 (best autoregulation).

**Figure 6.**
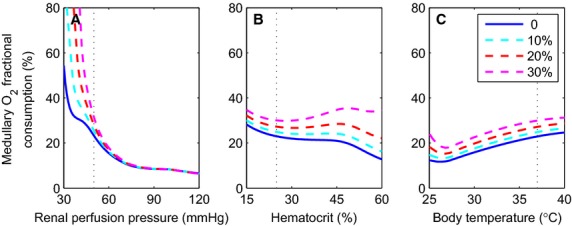
Renal oxygenation sensitivity during CPB rewarming. Medullary O_2_ fractional consumption as a function of renal perfusion pressure (A), hematocrit (B), and body temperature (C). For all simulations, arterial blood PO_2_ is 400 mmHg and RPP is nonpulsatile. Reference state is at RPP of 50 mmHg, hematocrit of 25%, and body temperature 37°C (denoted by dotted lines). The four curves in each of the panel correspond to differing degrees of MBF autoregulation (*b*).

We conducted simulations in which the CPB rewarming was used as the reference phase. In particular, reference RPP is 50 mmHg, hematocrit 25%, and body temperature 37°C. In three sets of simulations, we computed fractional medullary O_2_ consumption for a range of values of RPP and hematocrit. For each set of simulations, we also varied the degree of MBF autoregulation by setting *b* = 0, 10%, 20%, and 30%. Results are shown in Figure 6. The model predicts that a reduction in RPP has the most marked effect on medullary oxygenation. As previously noted, RPP during surgery on CPB often falls below the range of values that autoregulation can adequately compensate for (Brady et al. 2010). Thus, the model predicts that, with the most robust autoregulation of MBF, decreasing RPP to 30 mmHg (Brady et al. 2010), a value that is by no means atypical during surgery on CPB, decreases SNGFR, decreases medullary O_2_ delivery, and dramatically raises medullary O_2_ consumption to nearly 100% of O_2_ delivery. When MBF autoregulation is less robust (for example *b* = 30%), a similarly high fractional oxygen extraction can be obtained at RPP as high as ~45 mmHg.

### Impact of variations in temperature during the hypothermic phase of cardiopulmonary bypass

**Figure 7.**
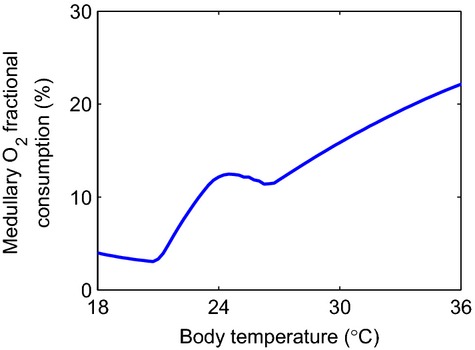
Fractional extraction of O_2_ in the medulla during the hypothermic phase of CPB, as a function of body temperature.

Our model assumes a temperature of 28°C in the hypothermic phase of cardiopulmonary bypass. However, routine procedures for cardiac surgical patients may be performed at higher temperatures of 32–34°C, whereas major aortic cases are often cooled to 18°C. Thus, we study the impact of variations in temperature during the hypothermic phase on medullary fractional oxygen extraction. Results are shown in Figure 7. The dependence of oxygen extraction fraction on temperature is nonmonotonic owing to the competing effects of higher tubular active NaCl reabsorption rate and increased medullary blood flow (thus O_2_ supply) as temperature increases. The fractional extraction of oxygen markedly increases as temperature is raised from 28°C (13%) to 34°C (20%), thereby rendering the medulla at a much higher risk to hypoxic injury. For comparison, the fractional extraction of oxygen was calculated to be 23% at 37°C.

## Discussion

The principal goal of this study was to determine the phase or phases of cardiac surgery on CPB during which the renal medulla is most susceptible to hypoxia, and the renal hemodynamic and functional factors that drive this susceptibility. Simulation results have identified the rewarming phase of CPB, in which medullary blood flow is low but medullary oxygen consumption remains high, as the phase in which the kidney is the most vulnerable to hypoxic injury (Fig. 3). The model predicts that during the rewarming phase of CPB, 22.9% of the medullary O_2_ supply is consumed. Since net medullary O_2_ supply exceeds consumption by a comfortable margin, it may not be immediately clear how such an effect might render the medulla susceptible to hypoxic damage. The explanation lies in the anatomy of the kidney: the spatial arrangement of nephrons and vessels has been found to be highly structured in a number of mammalian kidneys (Kriz 1981), including rats, mice, and human (Kriz 1967; Kriz et al. 1972; Kriz and Koepsell 1974; Jamison and Kriz 1982). The structural organization of the renal vasculature limits oxygen delivery to renal tissue, as discussed below.

In the outer medulla, vasa recta form tightly packed vascular bundles that appear to dominate the histotopography, especially in the inner stripe. Collecting ducts and thick ascending limbs are found distant from the bundles. The sequestration and countercurrent arrangement of the descending and ascending vasa recta preserves O_2_ delivery to the inner medulla (Chen et al. 2009). However, given the high metabolic demand of the thick ascending limbs, their separation from the oxygen‐carrying descending vasa recta subjects the thick limbs to risk of hypoxia. PO_2_ in the inner medulla is lower than in the outer medulla (Evans et al. 2013), but the thin ascending limbs in the inner medulla do not mediate significant active NaCl transport and thus have much lower oxygen demand. Thus, we focus our discussion on the outer medulla.

Measurements of PO_2_ in the renal outer medulla typically range from 20 to 30 mmHg (Dinour and Brezis 1991; Brezis et al. 1994; Liss et al. 1998; dos Santos et al. 2007; Palm et al. 2008). However, the structural organization of the tubules and vessels likely results in a substantial radial gradient in PO_2_. A recent study (Fry et al. 2014) using a detailed mathematical model of O_2_ transport in the renal medulla indeed predicts such a radial gradient in interstitial PO_2_, from as high as ~35 mmHg in the core of the vascular bundle, to as low as ~5 mmHg in the interbundle region. The model thick ascending limbs operate near anoxia, with average luminal PO_2_ as low as 2.5 mmHg in the inner stripe.

**Figure 8.**
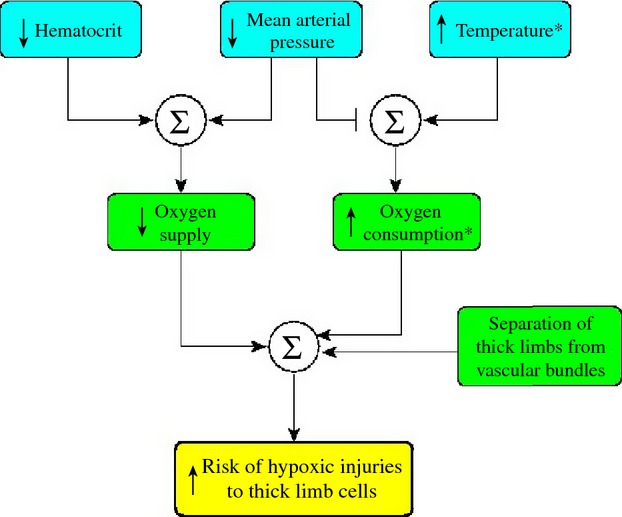
Pathway for the development of hypoxic injury. *Indicates effects during the CPB rewarming phase.

The results we present were computed using baseline model parameters, which implicitly assume conditions similar to the case of normothermia in the present study. When vascular inflow and hematocrit were lowered to simulate conditions in the rewarming phase of CPB, thereby lowering O_2_ supply, thick limb luminal PO_2_ fell to 1.25 mmHg (Fry et al. 2014). Simulations using the present model indicate that the risk of hypoxia can markedly increase even with a modest decrease in RPP during surgery (Fig. 6A). This result has a troubling implication, because of the technical difficulties in stabilizing perfusion pressure within a narrow range during CPB. In fact, during surgery perfusion pressure can fall to as low as 30 mmHg (Brady et al. 2010), which exposes the patients to serious risk of renal hypoxia. This pathway to hypoxic injuries is summarized in Figure 8.

The present model assumes the MBF is approximately a constant fraction of renal blood flow. However, measurements by Mattson et al. (Mattson et al. 1993) indicate that cortical blood flow is significantly better autoregulated than MBF as RPP varies. This implies that our model may overestimate MBF at low perfusion pressure, and underestimate the extent of medullary hypoxia.

A key mechanism by which CPB affects renal hemodynamics and oxygenation is through the effects of hypothermia, which include vascular tone, plasma viscosity, glomerular filtration, and metabolism. However, relevant data are sparse. Thus, to model kidney function during CPB, we have made assumptions based almost entirely on a single study, that by Broman and Källskog (Broman and Källskog 1995). Without supporting measurements from additional studies, it is unclear how robust those assumptions are. It is noteworthy that the model's key prediction, that the kidney is particularly vulnerable to hypoxic injury during the rewarming phase (as opposed to the hypothermic phase), depends rather critically on the kidney's metabolic demand, which is attributable in no small part to the active transport of the renal tubules. However, how tubular active transport varies as a function of body temperature has not been quantified, and we have made model assumptions based on urine flow and composition measured by Broman and Källskog (Broman and Källskog 1995) in hypothermic and normothermic rats. Given the critical role of metabolism in renal oxygen demand, more precise measurements would strengthen the model's prediction and yield a better understanding of the risk of AKI during different CPB phases.

## Conflict of Interest

None declared.

## Appendix

### Model Equations

The present model is an extension of a mathematical model of renal hemodynamic control previously developed by us (Sgouralis and Layton 2014). A schematic representation is shown on Figure 1. The model consists of a large set of equations and parameters, which can be found in (Sgouralis and Layton 2014) and the references therein. Below we summarize some of the key model equations, and highlight the modifications made to include the effects temperature and hemodilution that are not captured in (Sgouralis and Layton 2014).

#### Autoregulatory mechanisms

The afferent arteriole is the effector site of the myogenic mechanism and tubuloglomerular feedback (TGF). The model afferent arteriole segment consists of a series of smooth muscle cell models. Each smooth muscle model incorporates membrane potential, transmembrane ionic transports, cytosolic Ca^2+^ regulation, and muscle contraction. The rate of change in the membrane potential  of the i‐th smooth muscle is given by the sum of transmembrane currents

where *C*_*m*_ denotes the cell capacitance, and  denote transmembrane leak current, potassium current, calcium current, gap junctional current between smooth muscles, and gap junctional current between smooth muscle and the endothelium, respectively. The remaining currents,  and , arise from the operation of the myogenic response and TGF, described below.

With the myogenic response, a rise in intravascular pressure elicits a reflex constriction that generates a compensatory increase in vascular resistance, after an initial delay of *τ*_*m*_. The overall response time is faster for vasoconstriction than vasodilation (Loutzenhiser et al. 2002, 2004). To capture this response, the model assumes that the activity of nonselective cation channels is shifted by changes in intravascular pressure, such that the smooth muscle membrane depolarizes with increasing intravascular pressure and vice versa. This process is represented by the pressure‐dependent current  in Equation 9 given by

where  denotes the intravascular pressure. The rate constant *k*_MR_ at time t depends on the direction in which  is changing at an earlier time *t* – *τ*_*m*_. The target myogenic current  is assumed to be a sigmoidal function of the deviation of  from its baseline value .

The TGF signal is represented by the current  which is applied to the smooth muscles spanning the distal 60 *μ*m of the afferent arteriole. The current  is assumed to exhibit a sigmoidal dependence on the deviation of the intratubular macula densa [Cl^−^] from its operating point.

The myogenic response and TGF affect membrane potential , which in turns changes the opening probability of the voltage‐gated Ca^2+^ channels and the associated flux . Smooth muscle‐free cytosolic Ca^2+^ concentration, denoted *c*^*i*^ (in nmol/L), is assumed in equilibrium with the buffer and is related to  (given in mV/sec) by

where *k*_Ca_ is the first‐order rate constant for cytosolic calcium extrusion (Chen et al. 2011). We assume that afferent arteriole myocyte cytosolic [Ca^2+^] increases at lower temperature. This is achieved by decreasing afferent arteriole smooth muscle cytosolic Ca^2+^ extrusion rate with temperature (Broman and Källskog 1995) by assuming the following dependence on temperature

Temperature effects, here and below, are modeled by linear curves fitted to the available data at 37°C (reference temperature) and 28°C (Broman and Källskog 1995). Cytosolic [Ca^2+^] determines the phosphorylation level *ψ*^*i*^

which, in turn, determines the fraction of formed crossbridges, denoted *ω*^*i*^, according to

where *ω*^*i*^ determines the active wall tension of the smooth muscle cells, and thus the luminal radius of the afferent arteriole.

Efferent arteriole luminal radius is determined similarly, with the exception that the crossbridge formation value, *ω*_EA_, depends on temperature but not pressure, inasmuch as the efferent arteriole is myogenically inactive. We assume that *efferent arteriole muscle activation* increases with temperature (Broman and Källskog 1995); thus, we have

#### Renal blood flow

To compute vascular blood flow, we assume that the model afferent arteriole is connected in series to a pre‐afferent arteriolar resistor Ω_RA_ and a post‐afferent arteriolar resistor Ω_EA_. The pre‐afferent arteriolar resistance (in mmHg·sec/nL) is assumed to be a function of hematocrit, given by

The overall resistance of the model afferent arteriole is computed from the radius profile

where *L*_AA_ = 303 *μ*m is afferent arteriole length, and *μ*_AA_ denotes blood viscosity, which is a function of hematocrit

The representation of the impact of hemodilution on blood viscosity follows the approach of Pries et al. (Pries et al. 1992) (equation 9 therein). The component *μ*_plasma_ (in mPa·sec) is a function of temperature (Lim et al. 2010), given by

Postglomerular resistance is computed by

where *L*_EA_=303 *μ*m denotes the efferent arteriole length, and *μ*_EA_ is the postglomerular viscosity, given by

The evaluation of postglomerular hematocrit Ht_EA_ accounts for plasma loss due to filtration and is given by

We assume simple Poiseuille flow, so that arteriolar flow can be computed from the pressure drop along the afferent arteriole and the arteriolar resistance

where  is the afferent arteriole outflow pressure (see Sgouralis and Layton (2014) for the computation of *P*_AA_ along the afferent arteriole). Along the postglomerular vascular segment, blood flow is given by the difference between arteriolar flow and SNGFR, and is related to pressure drop and vascular resistance as follows

where *P*_GL_(*t*,*L*_GL_) is the glomerular outflow pressure (see below). Pressure at the end of the postglomerular segment is assumed to be 0 mmHg.

#### Glomerular filtration

The glomerulus is represented as a single capillary connecting the afferent and efferent arterioles. When RPP is pulsatile, a portion of the cardiac oscillations reaches the glomerular capillaries (Brenner et al. 1972; Aukland et al. 1977). In this case, filtration is driven by a waveform that consists of 3–4 wavelengths, based on the estimated transit time along the glomerular bed. Thus, cardiac oscillations are expected to be significantly damped by the process of glomerular filtration. To account for such damping, *Q*_AA_ and  are passed through a linear low pass filter

where the time constant *τ*_GL_ = 0.61 s is chosen according to the estimated transit time along the glomerular bed.

Let *Q*_GL_ and *C*_GL_ denote the plasma flow and plasma protein concentration profiles, respectively. Based on conservation of plasma mass, we have

where *y* denotes position along the glomerular capillary, *P*_neph_(*t*,0) denotes the proximal tubule inflow pressure and *π* denotes the colloid osmotic pressure (a function of *C*_GL_, see Sgouralis and Layton (2014)). *K*_f_ (in nL/min/mmHg) is the ultrafiltration coefficient, assumed to exhibit the following temperature dependence

*P*_GL_ is the hydrostatic blood pressure, assumed to decrease linearly along the capillary

where Δ*P*_GL_ (in mmHg) is the total pressure drop (i.e., *P*_GL_(*t*,0)–*P*_GL_(*t*,*L*_GL_)), and is assumed to depend on hematocrit

Plasma enters the glomerular capillary at a rate determined by hematocrit and arteriolar blood flow

SNGFR is given by the total fluid flux along the capillary

Oxygen delivery to the medulla is given by the total amount of free and bound oxygen (see Eq. 2 in main text).

Free and bound oxygen concentrations are given by the standard formulas (Popel 1989)

where *σ*_plasma_ (in nmol/L/mmHg) is oxygen solubility in plasma, which depends on temperature (Christoforides and Hedley‐Whyte 1969; Christoforides et al. 1969). Following the approach in Ref. (Battino et al. 1983), we assume that

In the expression for [HbO_2_] in Eq. (33), *c*_Hb_ = 4.75 mmol/L is concentration of heme in erythrocytes, and *s*_Hb_ is the saturation modeled by the Hill equation

The temperature dependence of *P*_50_ (in mmHg) is estimated experimentally in (Samaja et al. 1983) to be

#### Tubular transport

The model renal tubule includes a proximal tubule and a short loop of Henle. The model predicts intratubular pressure *P*_neph_, water flow *Q*_neph_, and [Cl^−^] profiles. Tubular water flow is pressure driven

where the temperature dependence of tubular fluid viscosity (in dyn·sec/m^2^) is given by

The proximal tubule and the initial segment of the descending limb of Henle's loop are water permeable. Taking the transmural water flux Φ_neph_ into account, pressure and flow are given by

Water flow entering the proximal tubule equals SNGFR. Transmural water flux is determined by SNGFR and renal perfusion pressure (RPP)

where  denotes time‐averaged RPP, and  is the baseline water flux profile, see Ref. (Sgouralis and Layton 2014). The factor *S*_GTB_(SNGFR) models glomerulotubular balance (Thomson and Blantz 2008; Thomson et al. Jan 2001)

where SNGFR is given in nL/min. Under physiologic conditions, fractional sodium reabsorption by the proximal tubule varies in tandem with SNGFR (Thomson and Blantz 2008; Thomson et al. Jan 2001). Cooling at 28°C yields almost no change in proximal tubule fractional reabsorption, despite the apparent reduction in SNGFR (Broman and Källskog 1995). To maintain a constant proximal tubule fractional reabsorption as temperature varies, we apply a temperature‐dependent factor *κ*_GTB_, given by

The second factor in Equation (40) , accounts for pressure natriuresis, where proximal tubular water reabsorption decreases with increasing perfusion pressure (Sgouralis and Layton 2014)

where RPP is in mmHg.

The model predicts luminal [Cl^−^] along the tubule, based on conservation of mass (Equation 3 in main text). The outflow [Cl^−^] is assumed to be at the site of the macula densa and is used to determine the TGF response. Maximum active transport rate *V*_max_ (nmol/cm^2^/sec) along the thick ascending limb depends on temperature as follows

This relation assumes negligible time lag between temperature increase and tubular transport upregulation.
